# Specific knowledge and resilience affect short-term outcome in patients following primary total hip arthroplasty

**DOI:** 10.1007/s00402-021-03967-0

**Published:** 2021-06-03

**Authors:** Alexander Bumberger, Katharina Borst, Madeleine Willegger, Gerhard M. Hobusch, Reinhard Windhager, Wenzel Waldstein, Stephan Domayer

**Affiliations:** 1 Department of Orthopedics and Trauma Surgery, Vienna General Hospital, Medical University of Vienna, Währinger Gürtel 18-20, 1090 Vienna, Austria; 2 Department of Trauma and Orthopaedic Surgery, BG Klinikum Unfallkrankenhaus Berlin gGmbH, Berlin, Germany; 3 Sonderkrankenanstalt Zicksee, Otto Pohanka Platz, 7161 Sankt Andrä am Zicksee, Austria

**Keywords:** Patient education, Specific knowledge, Resilience, Total hip arthroplasty, Total hip replacement, Total joint arthroplasty, Predicting outcome

## Abstract

**Purpose:**

The aim of the present study was to investigate the potential associations between specific knowledge, resilience and patient-reported outcome measures (PROMS) following primary total hip arthroplasty (THA).

**Methods:**

In a cross-sectional prospective study, consecutive patients following primary THA were included at a rehabilitation center. A novel knowledge score and the validated Connor Davidson Resilience Scale (CD-RISC) were utilized to assess patients’ specific knowledge and resilience, respectively. Additionally, patients completed a qualitative questionnaire regarding the information they had received. The Western Ontario and McMaster Universities Osteoarthritis Index (WOMAC), as well as the University of California and Los Angeles Score (UCLA) served as primary outcome measures. Stepwise multiple regression analysis was performed to identify potential predictors of outcome.

**Results:**

A total of 103 patients at a mean age of 67.5 years (SD 10.5, 38–88) were included in the analysis at a median of 55.5 days (IQR 43–81) following primary THA. The mean knowledge and resilience scores were 3.8 (SD 1.6, 0–7) and 69.5 (SD 18.5, 0–100), respectively. Forty-seven percent of patients were afraid of harming their prosthesis and these patients had up to 59% worse WOMAC scores (*p* < 0.001). WOMAC scores on admission to rehabilitation were predicted by resilience and knowledge scores (*R*^2^ = 0.106, *p* = 0.036). UCLA scores at the time of admission were predicted by knowledge scores (*R*^2^ = 0.078, *p* = 0.007).

**Conclusion:**

The present study demonstrated that patients with a feeling of uncertainty had an inferior short-term functional outcome following primary THA. Moreover, it could be shown that higher specific knowledge and resilience are associated with a better functional outcome according to validated PROMS. While these findings need to be prospectively validated in future studies, specific patient knowledge and resilience may have a direct impact on the outcome of primary THA.

## Introduction

Osteoarthritis (OA) of the hip and knee poses a major health burden and is ranked among the top contributors to global disability [1]. The incidence of primary total hip arthroplasty (THA) performed in Germany was recently predicted to increase by almost a third by the year of 2040 (2). THA is a safe and cost-effective procedure in patients with end-stage hip OA [2]. Despite its status as one of the most successful surgeries of the last decades [3], a significant number of patients remains unsatisfied after THA, with pain persistence and functional limitation representing the leading causes [4]. While there has been extensive research on surgical techniques and implant engineering, a few patient-related factors have recently attracted more attention. One aspect includes patients’ specific knowledge which was shown to be low in an arthroplasty population [5]. In this context, an interdisciplinary patient education program in total joint arthroplasty (TJA) was demonstrated to effectively decrease postoperative complications and the number of patients being discharged to post-acute care facilities [6]. Also, a one-on-one patient education session before THA or total knee arthroplasty (TKA) seems to significantly reduce length of hospital stay as compared to controls [7]. In contrast, an observational study evaluating patients from the Swedish hip arthroplasty register showed only minor effects of preoperative patient education on functional outcomes as assessed by patient-reported outcome measures (PROMS) [8]. However, as there is some considerable methodological heterogeneity of reported studies, the definite significance of patients’ individual knowledge about the procedure remains unknown and might be of underestimated relevance in TJA.

The potential significance of psychological factors regarding the outcome of THA is reflected by the results of a study by Scott et al., in which a higher mental component score predicted expectation fulfillment and patient satisfaction [9]. Mahdi et al. reported on the clinical outcomes of TKA, comparing patients with preoperative anxiety or depression to patients without. As patients showed substantial clinical improvements across all groups, the authors concluded that patients should not be precluded from TKA in case of preoperative anxiety or depression, although there were some minor differences in Knee Injury and Osteoarthritis Outcome Score (KOOS) sub-scores [10]. In a recent study by Al Salman et al., “difficult life events” such as the loss of a family member were associated with less activity tolerance according to Patient-Reported Outcome Measurement Information System Physical Function (PROMIS-PF) [11]. However, this was a very heterogeneous study group of patients presenting with any kind of lower extremity complaints. A few studies have identified resilience as a relevant predictor of outcome following joint replacement [12, 13]. While there is no commonly acknowledged definition of resilience, it has been described as “a stable trajectory of healthy functioning after a highly adverse event” and “a conscious effort to move forward in an insightful and integrated positive manner as a result of lessons learned from an adverse experience” by multidisciplinary panelists [14]. The authors further discussed ways to enhance resilience and concluded that fostering healthy family- and community environments is an essential factor [14]. In another study, a history of trauma has been demonstrated as a potential source of resilience in an arthroplasty population [15]. Magaldi et al. reported that higher preoperative resilience was a predictor for physical and mental health one year following surgery according to Patient-Reported Outcomes Measurement Information System (PROMIS-10) Global Health Assessment [13]. In another recent cross-sectional analysis of 140 patients by Lynskey et al., higher resilience was correlated with better patient-reported health status and satisfaction [12]. Although resilience might hardly be modifiable short-term, it may be an important aspect in the successful rehabilitation from a medical condition or surgery.

Striving for a better understanding concerning the significance of patients’ specific knowledge and resilience, the present study evaluated the association of these parameters with functional outcomes in a consecutive series of primary THA patients. We hypothesized that patients with higher specific knowledge and resilience scores would present with better functional outcomes at the time of admission to a regional rehabilitation center.

## Methods

Institutional review board approval by the local ethics committee was obtained and patients provided their written informed consent prior to participation. From December 2015 to December 2016 consecutive patients were included at a regional orthopedic rehabilitation center following primary THA at various orthopedic departments across Austria. Patients that had previously received TJA of any joint were excluded to obtain unbiased scores.

At the time of admission, age, sex, body mass index (BMI) and time elapsed since surgery were documented. All participants completed a questionnaire to assess their specific knowledge about THA, which was composed of seven questions concerning the surgical procedure and rehabilitation process, based on expert opinion and current literature (Table 1) [16, 17]. Most questions aimed to evaluate a basic understanding of THA as well as specific knowledge about important risks and how to prevent them that should usually be transferred to patients in a THA setting (e.g., avoiding deep hip flexion to minimize the risk of dislocation, etc.). The questionnaire was designed in a single best answer format with a score range from 0 to 7. The right answer to the question of which surgical approach was applied, was validated through review of the surgical reports.

**Table 1:** Questionnaire regarding specific knowledge

Q1: When is it safe for you to fully weight-bear?
Right after the procedure
6 months after the procedure
1 year after the procedure
I don’t know
Q2: Which surgical approach to the hip joint did your surgeon perform?
Anterior (frontal) approach
Between anterior (frontal) and lateral (sideways) approach (= anterolateral approach)
Lateral (sideways) approach
I don’t know
Q3: What kinds of movements are allowed at your current state of rehabilitation?
Flexing/bending [hip joint] up to 90°
Leg crossing
Deeply bending forward [hip joint]?
I don’t know
Q4: What is the most common complication in the first year following hip replacement?
Dislocation of the hip joint
Periprosthetic fracture (adjacent to the hip joint)
Loosening
I don’t know
Q5: What activities are allowed at your current state of rehabilitation?
All kinds of sports
Low-impact endurance sports (e.g. cycling, Nordic walking)
Resting the hip joint is favorable
I don’t know
Q6: When is it safe for you to ride a bicycle or go Nordic Walking?
6 weeks after the procedure
3 months after the procedure
6 months after the procedure
1 year after the procedure
Q7: What are the chances of not needing a revision surgery within 10 years?
90%
60%
50%
I don’t know

To assess patients' resilience, the 25-items Connor Davidson Resilience Scale (CD-RISC 25) was applied, which is a validated tool [18, 19] and was recently shown to correlate with patient satisfaction following THA and TKA [12]. The CD-RISC 25 has a maximum score of 100 and is available in German language. In the original publication, mean scores of around 80 were reported for the general population whereas patients with generalized anxiety disorder achieved lower scores of around 60 [18]. Since its first publication in 2003, numerous studies have applied the CD-RISC 25 in a variety of different populations, ranging from primary care patients [18, 20] to war veterans [21]. To the best of our knowledge, there are currently only two published studies that applied the CD-RISC in an arthroplasty population [12, 22].

To assess postoperative joint function, patients completed the Western Ontario and McMaster Universities Osteoarthritis Index (WOMAC), as well as the University of California and Los Angeles (UCLA) score. The WOMAC VA 3-series (“visual analog”) with a maximum score of 2400 was then normalized to a scale of 0–100. Higher WOMAC scores indicated worse joint function. Patients were asked to answer five additional qualitative questions regarding the information they were provided with during hospitalization and outpatient clinics for THA.

### Statistical analysis

Differences in demographics and PROMS between female and male patients, as well as differences in PROMS by answers given to qualitative questions were analyzed via independent samples t-tests. Stepwise linear regression analysis with multiple inputs was performed to determine predictive variables for functional outcome. WOMAC and UCLA scores served as outcome measures (dependent variable); age, sex, BMI on admission, time interval from surgery to admission for rehabilitation, knowledge scores and CD-RISC served as inputs (independent variables). Regression coefficients (b) for individual predictors are provided in the results section, as well as R^2^ as an indicator of model fit. The level of significance was set to *α* = 0.05. All calculations were performed in SPSS v. 25.0 (IBM, Armonk, New York, USA).

A post-hoc power analysis was performed using G*Power 3.1 (Heinrich Heine Universität, Düsseldorf, Germany). The resulting effect size f^2^ was 0.17 for the WOMAC and 0.12 for the UCLA score based on the predictor-outcome correlations and inter-predictor correlations. Considering our sample size of 103 patients and *α* = 0.05, the power of the current study to detect associations between predictor variables and outcome measures was 87.8% for WOMAC scores and 72.0% for UCLA scores, respectively.

## Results

A total of 103 patients at a mean age of 67.5 years (SD 10.5, 38–88) and a mean BMI of 29.0 kg/m^2^ (SD 4.8, 20–42) were included in the analysis at a median of 55.5 days (IQR 43–81) following primary THA. Male patients, comprising 48.5% of the participants, had a significantly higher BMI (30.0, *p* = 0.042) upon admission for rehabilitation than female patients. The mean knowledge and resilience scores were 3.8 (SD 1.6, 0–7) and 69.5 (SD 18.5, 0–100), respectively. No gender-specific differences were observed for knowledge and resilience scores. Patient age was negatively correlated with resilience scores (*r* = − 0.237, *p* = 0.017).

**Fig. 1:**
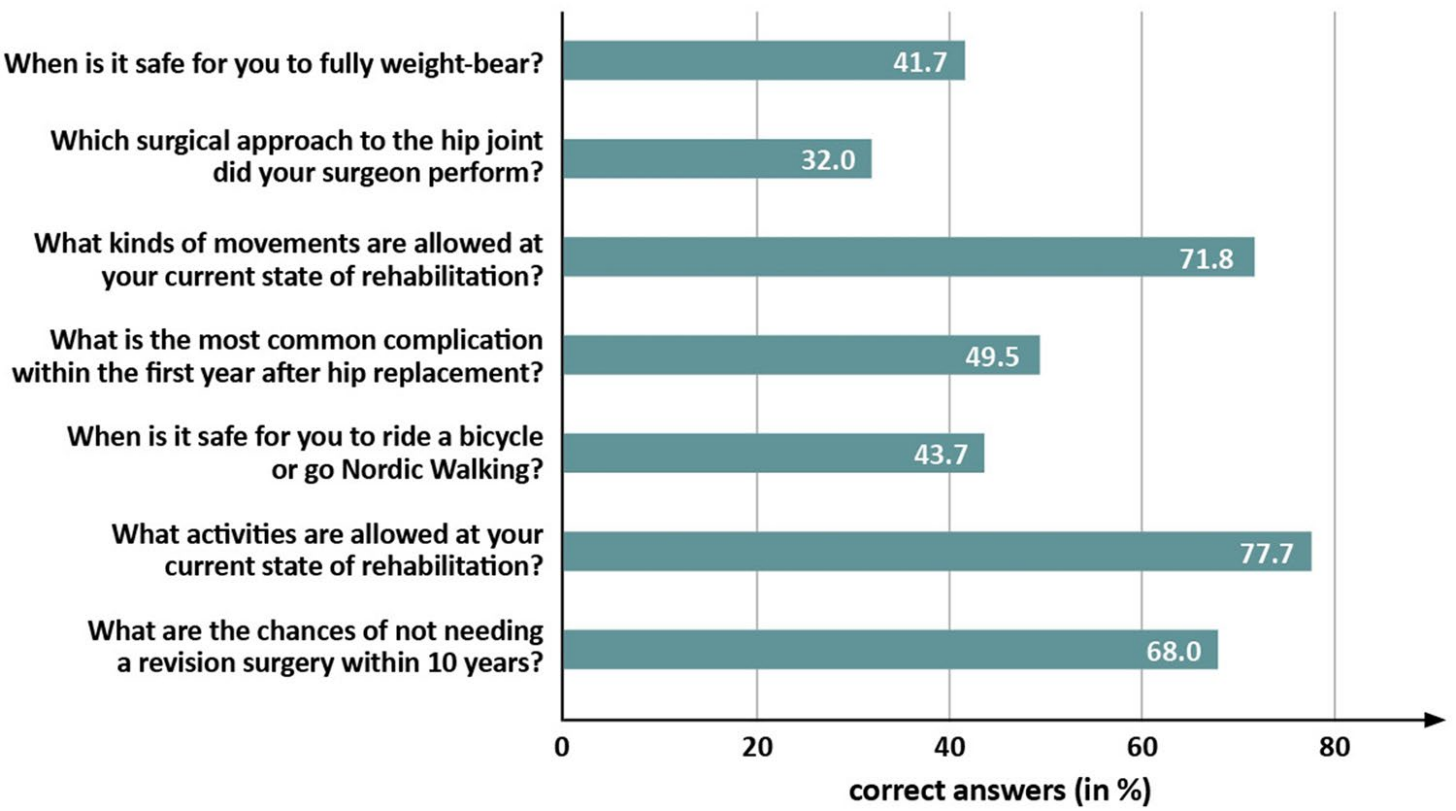
Percentage of correct answers given for each question on the knowledge questionnaire

**Fig. 2:**
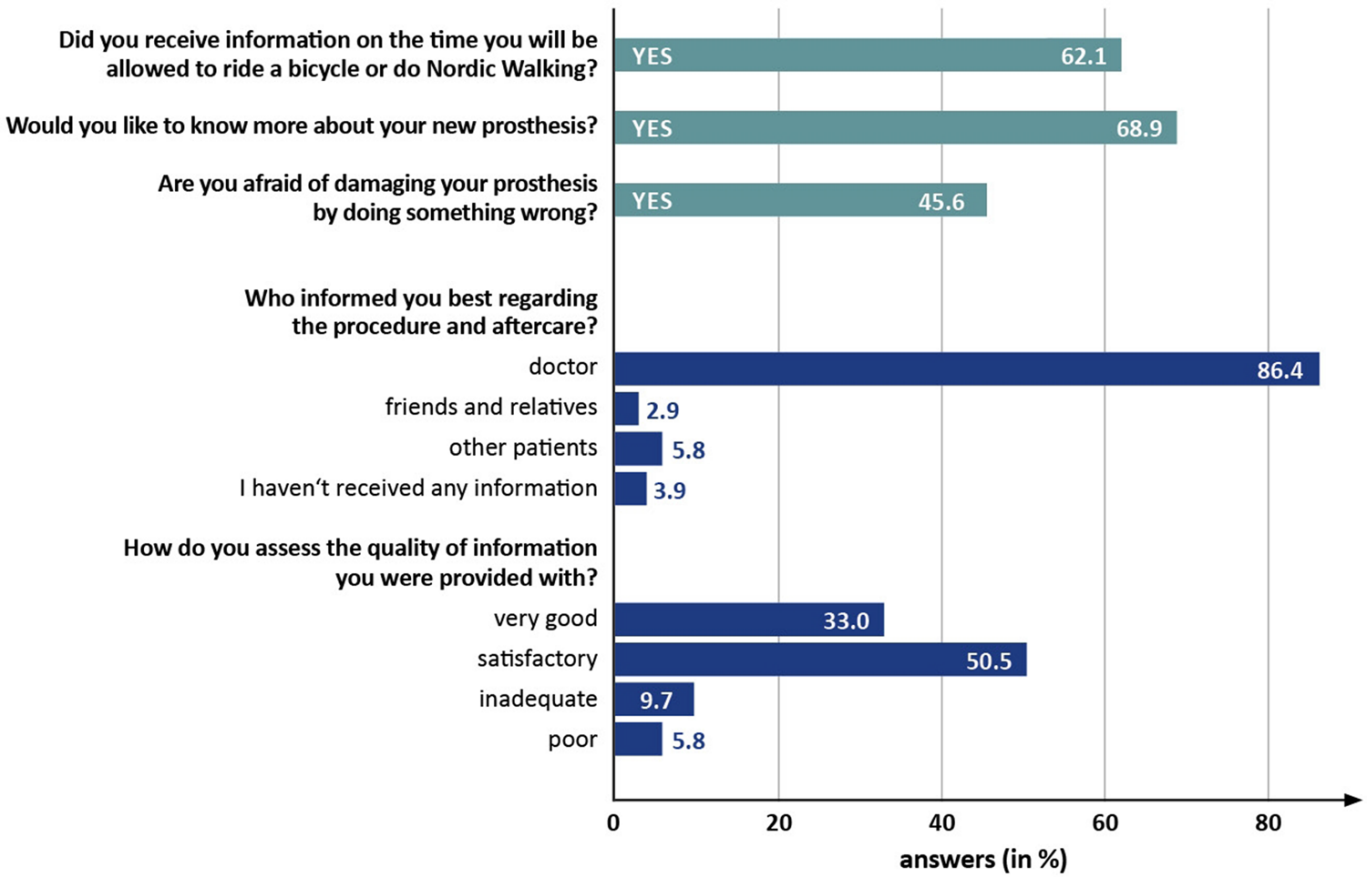
Percentage of answers given for each question on the qualitative questionnaire

The question “What activities are allowed at your current state of rehabilitation?” had the highest percentage of correct answers (78%) (Fig. 1). “Which surgical approach to the hip joint was performed by your surgeon?” was the question with the least percentage of correct answers (32%). Thirty-three percent assessed the quality of information they were provided with as “very good”; however, 51% rated the information as only “satisfactory” (Fig. 2). The remaining 16% found the information to be either “inadequate” (10%) or “poor” (6%). Forty-seven percent of the patients were afraid of harming their prosthesis and 69% were eager to learn more about the implant. Students’ *t*-test for independent samples demonstrated that patients with a feeling of uncertainty (afraid of harming their prosthesis) had up to 59% worse WOMAC scores (*p* < 0.001) and up to 15% worse UCLA scores (*p* = 0.016) as compared to patients without (Table 2).

**Table 2:** Functional scores by answers to qualitative questions

	Yes	No	*p*-value
Did you receive information on the time you will be allowed to ride a bicycle or do Nordic walking?			
WOMAC	21.6 (13.8)	23.5 (14.2)	0.51
UCLA	5.3 (1.5)	4.8 (1.8)	0.187
Would you like to know more about your new prosthesis?			
WOMAC	23.0 (14.6)	19.6 (12.2)	0.267
UCLA	5.2 (1.7)	5.0 (1.5)	0.761
Are you afraid of damaging your prosthesis by doing something wrong?			
WOMAC	27.9 (14.7)	17.5 (11.0)	< 0.001*
UCLA	4.7 (1.7)	5.5 (1.5)	0.016*

Independent samples *t*-test, two-sided, mean values (standard deviation)

*p < 0.05

**Fig. 3:**
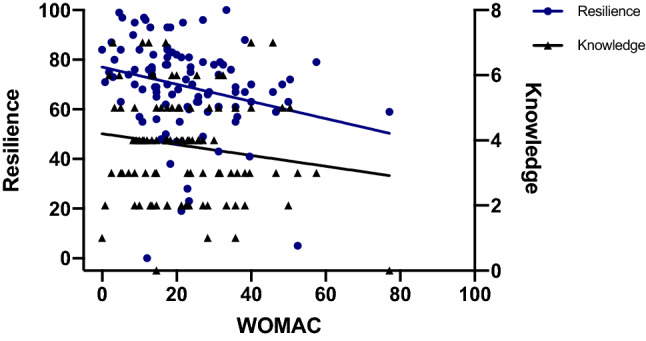
Scatterplots and regression lines for both knowledge and resilience scores with WOMAC as dependent outcome measure

Stepwise multiple linear regression analysis was performed for both WOMAC (Table 3) and UCLA (Table 4) scores as outcome parameters, and age, sex, BMI, days since surgery, knowledge score and resilience as independent variables. WOMAC scores on admission for rehabilitation were predicted best by resilience and knowledge scores (*p* = 0.006) (Fig. 3). UCLA scores at the time of admission were best predicted by knowledge scores (*p* = 0.007). Age, sex, BMI and days since surgery were not correlated with WOMAC or UCLA scores. The summarized results of Pearson’s bivariate correlation analysis are provided in Table 5.

**Table 3:** Multiple regression analysis (dependent variable: WOMAC)

	Unstandardized coefficients		Standardized coefficients	*T*	*p*
	Regression coefficient B	Std. error	Beta		
(Constant)	41.857	5.995		6.982	0.000
Resilience	− 0.168	0.073			
	0.229				
	− 2.314	0.023			
Knowledge	− 1.852	0.872	− 0.210	− 2.123	0.036
*R*^2^	0.106				
*R*^2^ adjusted	0.086				

Western Ontario and McMaster Universities Osteoarthritis Index (WOMAC)

**Table 4:** Multiple regression analysis (dependent variable: UCLA)

	Unstandardized coefficients		Standardized coefficients	*T*	*p*
	Regression coefficient B	Std. error	Beta		
(Constant)	3.946	0.440		8.970	0.000
Knowledge	0.289	0.104	0.279	2.782	0.007
*R*^2^	0.078				
*R*^2^ adjusted	0.068				

University of California and Los Angeles Activity Scale (UCLA)

**Table 5:** Correlations of independent variables and outcome measures

	Knowledge	Resilience	Age	Sex	BMI	Days
WOMAC	− 0.231 (0.012)*	− 0.248 (0.008)*	0.183 (0.038)	− 0.039 (0.353)	0.107 (0.151)	0.086 (0.204)
UCLA	0.279 (0.003)*	0.045 (0.332)	0.054 (0.304)	− 0.011 (0.458)	− 0.092 (0.188)	0.083 (0.212)

Pearson’s correlation coefficients (*p*-values)

*BMI* Body Mass Index, *WOMAC* Western Ontario and McMaster Universities Osteoarthritis Index, *UCLA* University of California and Los Angeles Score, *Days* days since surgery

*p < 0.05

## Discussion

To the best of our knowledge, this is the first study to demonstrate an association between patient knowledge, patient resilience and the postoperative functional outcome following primary THA. The results of this cross-sectional analysis showed that higher specific knowledge and resilience were associated with an improved short-term outcome following THA. We found that higher knowledge and resilience scores were associated with improved WOMAC scores at two months postoperatively. Also, an increased level of activity (UCLA) at this time was associated with higher knowledge scores. These findings add to the yet small body of literature, demonstrating significant associations between patient knowledge, psychological factors and PROMS following primary TJA [8, 22–24].

The mean WOMAC score of included patients was 22.3 at around two months following THA. This compares well to reported WOMAC scores of 18.4 and 13.0, at six months and one year following primary THA, respectively [25, 26]. Also, the mean CD-RISC score of 69.5 seems plausible, considering that mean scores around 80 are reported for the general population, and 60 for patients with generalized anxiety disorder. At a mean age of 67.5 years, the present cohort was slightly older at the time of surgery than those of previous studies. The mean postoperative UCLA activity score in the present study was 5.1 with no gender- or age-related differences. This finding is in line with previous studies reporting short-term UCLA scores of 4.5–6 [25, 27].

The qualitative questionnaire revealed that nearly half of the patients (47%) had a feeling of uncertainty to potentially harm their prosthesis during rehabilitation. While patients must be aware of restrictions like avoiding deep hip flexion following THA, they also need to have confidence in their implant to obtain satisfactory function. Interestingly, most patients (69%) were keen on receiving further information regarding their implant. The most remarkable finding though was an association between the feeling of uncertainty and inferior PROMS. Unlike other known modifiable risk factors, such as obesity and overall comorbidity [28], uncertainty of patients may be addressed with relatively little effort. The quality of provided information was assessed as good (33.0%) or satisfactory (50.5%) by most participants. However, no standardized information was provided, and quality therefore might have been inconsistent.

A concise questionnaire including seven multiple choice questions was applied to assess specific knowledge of participants. While this score has not been validated, it is thought to cover relevant aspects in terms of rehabilitation following THA. Specific knowledge was reported to be generally low in patients undergoing arthroplasty, pointing towards a potentially underestimated area of improvement [5]. Although there is no standardized way of assessing patients’ specific knowledge in THA, the results of the current study seem to support these findings, as patient knowledge was only moderate at an average score of 3.8 out of 7. Especially older age and lower formal educational attainments have been shown to be associated with worse knowledge [5]. In contrast, in the current study, higher age was not associated with significant alterations in knowledge scores. Only a few studies have investigated ways of improving patient knowledge in elective orthopedic procedures. Among these, in a randomized-controlled trial (RCT) by Eschalier et al. [29], educational booklets have significantly improved knowledge in patients awaiting TKA. Despite this difference, there were no significant variations in secondary outcome measures, including percentage of patients being discharged to home and patient satisfaction. However, with a study population of only 42 patients, this study was most likely underpowered to detect the effect of this intervention. Pelt et al. demonstrated that a comprehensive patient education can actually reduce the number of patients being discharged to post-acute care facilities, thereby reducing costs and complication rates [6]. There was a 20% decline in the number of patients being discharged to post-acute care facilities following the consecutive implementation of a comprehensive educational program for patients undergoing THA. Wallis et al. concluded in a meta-analysis that preoperative exercise and education programs can improve functional outcome following THA [30]. These findings are supported by our own data that revealed a significant association between higher knowledge and superior functional outcome according to WOMAC and UCLA scores. SooHoo et al. reported a minimal clinically important difference (MCID) in UCLA scores of 0.92 in an arthroplasty population [31]. Hence, a substantial improvement in knowledge scores could actually translate into clinically relevant improvements considering the regression coefficient of 0.289 in the present study. Based on our data, this would result in a theoretic threshold of a 3.18-point improvement in knowledge scores to achieve a MCID according to the UCLA activity scores (3.18 * 0.289 = 0.92). Regarding WOMAC scores, a MCID of 10 was reported for the overall score [32]. Considering the regression coefficient of -1.852, a patient would therefore require a minimum of 5.4-point increase in the knowledge score (5.4 * − 1.852 = 10) to yield clinically relevant improvements. In summary, our results support evidence attributing a significant role to patient knowledge and education regarding the functional short-term outcome of THA.

Although using different resilience scales and outcome measures, our results support the findings of previous studies [9, 12, 13], in that higher resilience is associated with improved patient-reported outcome following primary THA. In detail, multiple regression analysis demonstrated a decline in WOMAC scores of 0.168 by every additional point on the CD-RISC. Therefore, at an average postoperative WOMAC score of 22.3, a 10-point increase in CD-RISC would have accounted for an 8% decline in WOMAC, indicating an improved outcome. However, it must be acknowledged that the potential improvements in WOMAC scores due to increased resilience might eventually stay below the threshold for a MCID which has been reported at 10 points for the overall WOMAC score [32]. Hence, the present study does not suggest resilience as determining factor for an improved function according to the WOMAC score. However, it introduces resilience as a variable affecting PROMS following primary THA.

The reported coefficients of determination (*R*^2^) of the linear regression analyses were rather low, indicating that there is a considerable variance of observed data that cannot be explained by the regression model. However, knowledge and resilience scores performed better in predicting postoperative PROMS as compared to other demographic parameters, including age, sex, BMI and time since surgery, which were not significantly correlated with outcome measures. With respect to the 87.8% power to detect significant correlations, we are confident that there is no type II error, as far as WOMAC scores are concerned. The study was slightly underpowered to detect correlations between predictors and UCLA scores which might account for the discrepancy that resilience was correlated with WOMAC, but not with UCLA scores, as opposed to knowledge scores.

Looking at the present data and literature available, it can be suggested that a more comprehensive treatment process with respect to both constitutional and psychological factors is an effective way to further optimize the outcome of primary THA. Also, such an educational process could provide an opportunity to adjust patients’ expectations, which are a potential source of dissatisfaction if unmet [9, 33].

This study has several limitations. Patients were included upon admission to an orthopedic rehabilitation center, posing a potential selection bias in favor of patients with inferior outcome. However, orthopedic rehabilitation following TJA is still very common in Austria regardless of age and functional outcome. Due to the lack of an established score to assess patients’ knowledge in THA, a new non-validated score was utilized. Hence, the results regarding the associations between knowledge, resilience and PROMS must only be interpreted as hypothesis-generating findings. Patient education was not standardized as THA patients from various orthopedic institutions were included. Furthermore, preoperative joint function and early postoperative pain level- which are known independent predictors of outcome- were not evaluated.

## Conclusion

The present study demonstrated that patients with a feeling of uncertainty had an inferior short-term functional outcome following primary THA. Moreover, it could be shown that higher specific knowledge and resilience are associated with a better functional outcome according to validated PROMS. While these findings need to be prospectively validated in future studies, specific patient knowledge and resilience may have a direct impact on the outcome of primary THA.

## Data Availability

All data and materials comply with field standards.

## References

[1] Cross M, Smith E, Hoy D, Nolte S, Ackerman I, Fransen M (2014). The global burden of hip and knee osteoarthritis: estimates from the global burden of disease 2010 study. Ann Rheum Dis.

[2] Daigle ME, Weinstein AM, Katz JN, Losina E (2012). The cost-effectiveness of total joint arthroplasty: a systematic review of published literature. Best Pract Res Clin Rheumatol.

[3] Learmonth ID, Young C, Rorabeck C (2007). The operation of the century: total hip replacement. Lancet.

[4] Halawi MJ, Jongbloed W, Baron S, Savoy L, Williams VJ, Cote MP (2019). Patient dissatisfaction after primary total joint arthroplasty: the patient perspective. J Arthroplasty.

[5] Billon L, Décaudin B, Pasquier G, Lons A, Deken-Delannoy V, Germe A-F (2017). Prospective assessment of patients’ knowledge and informational needs and of surgeon-to-patient information transfer before and after knee or hip arthroplasty. Orthop Traumatol Surg Res.

[6] Pelt CE, Gililland JM, Erickson JA, Trimble DE, Anderson MB, Peters CL (2018). Improving value in total joint arthroplasty: a comprehensive patient education and management program decreases discharge to post-acute care facilities and post-operative complications. J Arthroplasty.

[7] Yoon RS, Nellans KW, Geller JA, Kim AD, Jacobs MR, Macaulay W (2010). Patient education before hip or knee arthroplasty lowers length of stay. J Arthroplasty.

[8] Torisho C, Mohaddes M, Gustafsson K, Rolfson O (2019). Minor influence of patient education and physiotherapy interventions before total hip replacement on patient-reported outcomes: an observational study of 30,756 patients in the Swedish hip arthroplasty register. Acta Orthop.

[9] Scott CEH, Bugler KE, Clement ND, MacDonald D, Howie CR, Biant LC (2012) Patient expectations of arthroplasty of the hip and knee. J Bone Joint Surg Br 94B:974–981. 10.1302/0301-620X.94B7.28219 10.1302/0301-620X.94B7.28219 22733956

[10] Mahdi A, Hälleberg-Nyman M, Wretenberg P (2020). Preoperative psychological distress no reason to delay total knee arthroplasty: a register-based prospective cohort study of 458 patients. Arch Orthop Trauma Surg.

[11] Al Salman A, Khatiri MZ, Cremers T, Ring D, Thomas JE, Fatehi A (2020). Difficult life events affect lower extremity illness. Arch Orthop Trauma Surg.

[12] Lynskey SJ, Ling F, Greenberg AM, Penny-Dimri JC, Sutherland AG (2020). The influence of patient resilience and health status on satisfaction after total hip and knee arthroplasty. Surgeon.

[13] Magaldi RJ, Staff I, Stovall AE, Stohler SA, Lewis CG (2019). Impact of resilience on outcomes of total knee arthroplasty. J Arthroplasty.

[14] Southwick SM, Bonanno GA, Masten AS, Panter-Brick C, Yehuda R (2014) Resilience definitions, theory, and challenges: interdisciplinary perspectives. Eur J Psychotraumatol. 10.3402/ejpt.v5.25338 10.3402/ejpt.v5.25338 PMC4185134 25317257

[15] Cremeans-Smith JK, Greene K, Delahanty DL (2015). Trauma history as a resilience factor for patients recovering from total knee replacement surgery. Psychol Health.

[16] Australian Orthopaedic Association National Joint Replacement Registry (AOANJRR) (2018) Hip, Knee & Shoulder Arthroplasty: 2018 Annual Report. Adelaide: AOA

[17] Dargel J, Oppermann J, Brüggemann G-P, Eysel P (2014). Dislocation following total hip replacement. Dtsch Arztebl Int.

[18] Connor KM, Davidson JRT (2003). Development of a new resilience scale: the connor-davidson resilience scale (CD-RISC). Depress Anxiety.

[19] Campbell-Sills L, Stein MB (2007). Psychometric analysis and refinement of the connor–davidson resilience scale (CD-RISC): Validation of a 10-item measure of resilience. J Trauma Stress.

[20] Wingo AP, Wrenn G, Pelletier T, Gutman AR, Bradley B, Ressler KJ (2010). Moderating effects of resilience on depression in individuals with a history of childhood abuse or trauma exposure. J Affect Disord.

[21] Green KT, Hayward LC, Williams AM, Dennis PA, Bryan BC, Taber KH, et al. Examining the factor structure of the Connor-Davidson Resilience Scale (CD-RISC) in a post-9/11 U.S. military veteran sample. Assessment 2014;21:443–451. 10.1177/1073191114524014 10.1177/1073191114524014 PMC4147024 24586090

[22] Bumberger A, Borst K, Hobusch GM, Willegger M, Stelzeneder D, Windhager R (2021). Higher patient knowledge and resilience improve the functional outcome of primary total knee arthroplasty. Wien Klin Wochenschr.

[23] Moyer R, Ikert K, Long K, Marsh J (2017). The value of preoperative exercise and education for patients undergoing total hip and knee arthroplasty: a systematic review and meta-analysis. JBJS Rev.

[24] Bay S, Kuster L, McLean N, Byrnes M, Kuster MS (2018). A systematic review of psychological interventions in total hip and knee arthroplasty. BMC Musculoskelet Disord.

[25] Postler AE, Beyer F, Wegner T, Lützner J, Hartmann A, Ojodu I (2017). Patient-reported outcomes after revision surgery compared to primary total hip arthroplasty. Hip Int.

[26] Rogers BA, Alolabi B, Carrothers AD, Kreder HJ, Jenkinson RJ (2015) Can the pre-operative Western Ontario and McMaster score predict patient satisfaction following total hip arthroplasty? Bone Joint J 97-B:150–153. 10.1302/0301-620X.97B2.34718 10.1302/0301-620X.97B2.34718 25628274

[27] Graves SC, Dropkin BM, Keeney BJ, Lurie JD, Tomek IM (2016). Does surgical approach affect patient-reported function after primary tha?. Clin Orthop Relat Res.

[28] Paxton EW, Inacio MCS, Singh JA, Love R, Bini SA, Namba RS (2015) Are there modifiable risk factors for hospital readmission after total hip arthroplasty in a US healthcare system? Clin Orthop Relat Res 473:3446–3455. 10.1007/s11999-015-4278-x 10.1007/s11999-015-4278-x PMC4586234 25845947

[29] Eschalier B, Descamps S, Pereira B, Vaillant-Roussel H, Girard G, Boisgard S (2017). Randomized blinded trial of standardized written patient information before total knee arthroplasty. PLoS ONE.

[30] Wallis JA, Taylor NF (2011). Pre-operative interventions (non-surgical and non-pharmacological) for patients with hip or knee osteoarthritis awaiting joint replacement surgery—a systematic review and meta-analysis. Osteoarthritis Cartilage.

[31] SooHoo NF, Li Z, Chenok KE, Bozic KJ (2015). Responsiveness of patient reported outcome measures in total joint arthroplasty patients. J Arthroplasty.

[32] Clement ND, Bardgett M, Weir D, Holland J, Gerrand C, Deehan DJ (2018). What is the minimum clinically important difference for the WOMAC index after TKA?. Clin Orthop Relat Res.

[33] Mancuso CA, Salvati EA, Johanson NA, Peterson MG, Charlson ME (1997). Patients’ expectations and satisfaction with total hip arthroplasty. J Arthroplasty.

